# Overexpression of the IL‐23/IL‐17A Axis and Their Receptors (IL‐23R and IL‐17RA) in Gingival Tissue of Patients With Periodontitis

**DOI:** 10.1155/ijod/8893504

**Published:** 2026-01-13

**Authors:** Sonia I. Vázquez-Jiménez, Ruth Rodríguez-Montaño, Vianeth M. Martínez-Rodríguez, Juan M. Guzmán-Flores, Ana L. Zamora-Perez, Susana del Toro Arreola, Celia Guerrero-Velázquez

**Affiliations:** ^1^ University Center of Los Altos, University of Guadalajara, Tepatitlán de Morelos, Jalisco, Mexico, udg.mx; ^2^ Institute for Research in Dentistry, Department of Comprehensive Dental Clinics, University Center for Health Sciences, University of Guadalajara, Guadalajara, Jalisco, Mexico, udg.mx; ^3^ Department of Health and Disease as an Individual and Collective Process, University Center of Tlajomulco, University of Guadalajara, Tlajomulco de Zúñiga, Jalisco, Mexico, udg.mx; ^4^ Specialty in Periodontology, Department of Comprehensive Dental Clinics, University Center for Health Sciences, University of Guadalajara, Guadalajara, Jalisco, Mexico, udg.mx; ^5^ Institute of Biosciences Research, University Center of Los Altos, University of Guadalajara, Tepatitlán de Morelos, Jalisco, Mexico, udg.mx; ^6^ Institute for Research in Chronic-Degenerative Diseases, Department of Molecular Biology and Genomics, University Center for Health Sciences, University of Guadalajara, Guadalajara, Jalisco, Mexico, udg.mx

**Keywords:** interleukin-17A, interleukin-17A receptor, interleukin-23, interleukin-23 receptor, periodontitis

## Abstract

**Background:**

Periodontitis is a chronic immunoinflammatory disease involving various components that affect the tissues surrounding the tooth. The host immune response to the presence of periodontopathogenic microorganisms activates several cytokine systems involved in alveolar bone resorption in periodontitis; among them is the receptor activator of nuclear factor‐κB (RANK)/receptor activator of nuclear factor‐κB ligand (RANKL) system, which depends on the interleukin (IL)‐23/IL‐17 axis. There is a tendency toward increased IL‐23, IL‐17, and IL‐17 receptor (IL‐17RA) and a discrepancy in IL‐23 receptor (IL‐23R) in gingival tissue (GT) of patients with periodontitis. Therefore, the aim of this study was to quantify the expression of the IL‐23/IL‐17A axis using the western blotting (WB) technique in GT samples from patients with periodontitis.

**Materials and Methods:**

This cross‐sectional study included 49 subjects: 25 healthy subjects and 24 subjects with periodontitis. GT samples were collected during periodontal surgery. WB was used to evaluate the levels of IL‐23, IL‐17A, IL‐23R, and IL‐17RA.

**Results:**

We found a significant increase in IL‐23, IL‐17A, IL‐23R, and IL‐17RA protein levels in the periodontitis group compared with the healthy group; we also detected bands with unexpected molecular weights for both receptors. Moreover, we found a significant positive correlation between IL‐23 and IL‐17A with both receptors, while IL‐23, IL‐17A, IL‐23R, and IL‐17RA correlated positively with two periodontal clinical parameters, namely the clinical attachment level and the percentage of radiographic bone loss (%RBL).

**Conclusion:**

In this study, we detected overexpression of IL‐23, IL‐17A, and their receptors in GT of patients with periodontitis, confirming that the IL‐23/IL‐17A axis is involved in periodontal disease.

## 1. Introduction

Periodontal disease is the second most important oral pathology affecting the world’s population. The World Health Organization has declared that periodontitis is in its most advanced stage and affects almost 10% of the population [1]. Periodontitis is a condition involving immunoinflammatory components that cause alterations in the tissues surrounding the tooth, causing loss of gingival attachment, alveolar bone loss, and the development of periodontal pockets, leading to tooth loss [2]. Periodontitis involves multiple etiological factors, so its development and progression depend primarily on the host’s immune response, bacterial dysbiosis, genetic variations, and environmental factors that modify inflammation and its evolution [3].

In this regard, the host’s immune response to periodontopathogenic bacteria activates several cytokine systems, including the interleukin (IL)‐23/IL‐17A axis. In this context, periodontal bacteria are known to stimulate dendritic cells (DCs) present in gingival tissue (GT) to secrete proinflammatory cytokines, such as IL‐23, which binds to its receptor (IL‐23R) expressed on T helper 17 (Th17) cells [4] to induce the synthesis of various cytokines such as IL‐17A and receptor activator of nuclear factor‐κB ligand (RANKL). Moreover, IL‐17A binds to its receptor (IL‐17RA), which is expressed on fibroblasts and triggers the release of RANKL. The synthesized RANKL binds to its receptor, activating nuclear factor‐κB (RANK), which is expressed on the membrane of osteoclast precursors, activating their differentiation and initiating with the process of alveolar bone resorption [5]. Studies have reported that RANKL is upregulated in patients with periodontitis [6], and one of the pathways for RANK/RANKL activation is the IL‐23/IL‐17A axis [7], which plays an important role in the immunopathology of periodontitis.

Several studies have analyzed the levels of IL‐23 and IL‐17, as well as their receptors (IL‐23R and IL‐17R), during different stages of periodontal disease and in various types of biological samples, including saliva [8], serum [9], plasma [10, 11], gingival crevicular fluid (GCF) [12], and GT [11, 13–18]. It is worth mentioning that in GT samples, several authors, including our group, have found elevated levels of IL‐23 and IL‐17 in patients with periodontitis using the polymerase chain reaction (PCR) technique, enzyme‐linked immunosorbent assay (ELISA), and immunohistochemistry [11, 13, 14, 16].

At protein level, we demonstrated an increase in IL‐17RA and, conversely, a decrease in IL‐23R in GT from patients with periodontitis compared to healthy subjects using the ELISA technique [11, 15]. In contrast, at the gene expression level, Ohyama et al. [16] demonstrated an increase in IL‐23R at the messenger RNA level in sites with periodontal lesions. However, Trumet et al. [18] found no significant differences when analyzing IL‐23R by immunofluorescence in both study groups. It should be noted that both IL‐23R and IL‐17RA have several isoforms [19–22], which may have different biological functions in some pathologies [23].

Although western blotting (WB) is a semi‐quantitative technique [24], it can reveal protein expression trends and elucidate the discrepancy of IL‐23R in GT in periodontitis. Therefore, the aim of this study was to evaluate the expression of the IL‐23/IL‐17A axis, as well as its receptors, using the WB technique, which can detect not only the canonical forms of the IL‐23/IL‐17A axis and its receptors in GT from patients with periodontitis, but also bands of different molecular weights.

## 2. Materials and Methods

### 2.1. Ethical Approval and Informed Consent

The present cross‐sectional study was approved by the Research Ethics and Biosafety Committees of the University of Guadalajara with the official number CI‐01622. The study’s objective was explained to all participants, and their written informed consent was obtained, which was prepared following the guidelines of the Declaration of Helsinki 2013 of the World Medical Association.

### 2.2. Sample Size

The sample size was calculated by analyzing the mean and standard deviations of previous studies concerning IL‐23 and IL‐17A levels in periodontitis, performed by Johnson et al. [13] and Chitrapriya et al. [17] to obtain a total of *n* = 24 subjects per study group. A non‐probabilistic method of collection by consecutive selection and analysis of single samples was used.

### 2.3. Study Subjects

Forty‐nine subjects were recruited and attended the Specialized Clinic in Periodontics of the University of Guadalajara from April 2022 to June 2024. All subjects were systemically healthy, not taking antibiotics or anti‐inflammatory drugs, and had not previously received periodontal treatment. Individuals with gingivitis, systemic disease, smokers, pregnant women, and subjects taking medication were excluded. Individuals were selected by a non‐probabilistic method of consecutive collection. Medical and dental histories were collected for each participant, followed by diagnosis and classification of periodontal status according to the new classification of periodontal diseases [25]. Clinical examination was performed with the collaboration of a calibrated specialist using a North Carolina UNC‐15 periodontal probe (Hu‐Friedy, Chicago, IL, USA). Six measurements were taken of each tooth present in each participant’s oral cavity, and the clinical parameters of clinical attachment loss (CAL), probing depth (PD), percentage bleeding on probing (BoP%), and percentage radiographic bone loss (RBL%) were evaluated. Subsequently, the subjects were assigned into two study groups:

### 2.4. Healthy Subjects (*n* = 25)

This group included periodontally healthy subjects from the periodontology clinic to come for an esthetic procedure of crown lengthening surgery; participants had no clinical inflammation (PD ≤ 3 mm, CAL ≤ 1 mm, BoP% < 10%, and no evidence of RBL%) [26].

### 2.5. Patients With Periodontitis (*n* = 24)

This group included patients diagnosed with periodontitis classified as Stage III or IV requiring surgery, who had clinical parameters of CAL ≥ 5 mm, PD ≥ 6 mm, BoP% ≥ 10%, periodontal pockets in more than 30% of the oral cavity, and radiographic evidence of RBL% [27].

### 2.6. GT Collection

The GT sample was collected during periodontal surgery, where epithelial and connective tissue was taken from the periodontal pocket. Tissue samples were arranged in 1.5 mL microtubes on frappé ice at 4°C and transported to the laboratory, where they were stored at −80°C in an ultrafreezer until analyzed. Samples that did not show an adequate amount of total protein by Bradford method and samples that did not express constitutive β‐actin protein were excluded.

### 2.7. Protein Extraction

Total protein was extracted by the micronization process. The collected GT samples were left at room temperature to thaw, then liquid nitrogen vapor was used to crystallize them, and they were crushed by compression until pulverized. The obtained triturate was placed in 200 μL RIPA buffer (Sigma, St. Louis, MO, USA) combined with a protease inhibitor (cOmplete, Roche Diagnostic GmbH, Indianapolis, IN, USA) at 4°C for 20 min, and then homogenized in a disruptor at 4°C for 20 min. The concentrate was centrifuged for 10 min at 12,000 rpm, and the supernatant obtained was collected and frozen at −80°C until analysis.

### 2.8. Total Protein Quantification

The total protein concentration in the GT supernatant was quantified using the Bradford method. This procedure involved reading the absorbance at 595 nm with a microplate spectrophotometer (FlexA‐200, Allsheng Instruments Co., Ltd., Hangzhou, Zhejiang, China) and determining the concentration based on a bovine serum albumin standard curve (Sigma, River Edge, NJ, USA). The concentration is expressed in µg/µL.

### 2.9. WB

Twenty micrograms of total protein obtained from GT was mixed with 10 μL of loading buffer and heated at 90°C for 10 min to denature the proteins, then loaded onto an 8%–12% sodium dodecyl sulfate‐polyacrylamide gel (SDS‐PAGE). Protein was separated by electrophoresis on a Mini Trans‐Blot Cell system (Bio‐Rad Laboratories, Inc., Hercules, CA, USA) at 80–120 V and 4°C.

Once the protein was separated, it was transferred to a polyvinylidene difluoride (PVDF) membrane (Bio‐Rad Laboratories, Inc.) with a Mini Trans‐Blot Cell system overnight at 30 V and 4°C. After completion of the electrophoretic transfer, the membranes were blocked with 3% nonfat dry milk to avoid non‐specific binding of the proteins.

The membranes were then incubated with the appropriate primary antibody overnight at 4°C: mouse monoclonal anti‐human IL‐17 (G‐4) IgG2B diluted 1:500 (sc‐374218, Santa Cruz Biotechnology, Santa Cruz, CA, USA), anti‐human IL‐23 (c‐3) monoclonal mouse IgG1 diluted 1:500 (sc‐271279, Santa Cruz Biotechnology), anti‐human IL‐17RA (D1Y4C) monoclonal rabbit IgG diluted 1:1000 (12661, Cell Signaling Technology, Danvers MA, USA), anti‐human IL‐23R (A48090) polyclonal rabbit IgG diluted 1:300 (Antibodies.com, Cambridge, UK), and β‐actin (C4) mouse monoclonal IgG1k diluted 1:1000 (sc‐47778, Santa Cruz Biotechnology).

The next day, the membranes were incubated with their respective mouse or rabbit secondary antibody conjugated to horseradish peroxidase (HRP) at 37°C for 1 h:goat anti‐mouse IgG‐HRP diluted 1:3000 (sc‐2005, Santa Cruz Biotechnology) and goat anti‐rabbit IgG‐HRP diluted 1:3000 (sc‐2004, Santa Cruz Biotechnology).

Finally, chemiluminescence detection was performed with the Immobilon Western kit (Millipore Corporation, Billerica, MA, USA). Immune detection and densitometric analysis were performed using a ChemStudio UVP chemiluminescence imaging system (Analytik Jena, Jena Germany). The density of the bands of these ILs and their receptors was determined by the expression of β‐actin in area relative units (ARUs).

### 2.10. Statistical Analysis

A chi‐square test was used to identify differences in sex frequency. The data distribution was evaluated using the Shapiro–Wilk test for small samples. When an abnormal distribution of the data was present, the nonparametric Mann–Whitney *U* test was used to compare the medians between the study groups. Spearman correlation coefficients were calculated to determine the correlations between protein expression and clinical parameters. SPSS Statistics Version 22.0 software (IBM Corp., Armonk, NY, USA) was used for statistical analysis. A *p*‐value ≤ 0.05 was considered significant.

## 3. Results

### 3.1. Demographic Characteristics and Clinical Parameters

The sociodemographic variables and clinical characteristics are presented as the mean ± standard deviation, while the stage and grade parameters are presented as a percentage. Of the 49 study subjects, the majority were female. The patients with periodontitis were significantly older than the healthy subjects. We found that patients with periodontitis presented a significant increase in CAL, BoP%, and PD compared with the healthy subjects. Of note, RBL% is a parameter for the classification of patients with periodontitis, so it is not available for the healthy subjects (Table 1).

**Table 1:** Demographic characteristics and clinical parameters.

Parameter	HS	P
Sex M/F	7/18	11/13
Age (years)	32.60 ± 12.79	49.08 ± 9.60 ^∗^
PD (mm)	1.79 ± 0.84	4.20 ± 1.20 ^∗^
CAL (mm)	1.15 ± 0.62	4.76 ± 1.07 ^∗^
BoP%	1.55 ± 1.38	41.46 ± 21.27 ^∗^
RBL%	–	63.51 ± 18.28
Stage III *n* (%)	–	16 (66.7)
Stage IV *n* (%)	–	8 (33.3)
Grade A *n* (%)	–	1 (4.2)
Grade B *n* (%)	–	13 (54.2)
Grade C *n* (%)	–	10 (41.6)

*Note:* Data are presented as mean and standard deviation or percentage. BoP%, percentage of bleeding on probing; RBL%, radiographic bone loss.

Abbreviations: CAL, clinical attachment loss; HS, healthy subjects; P, periodontitis; PD, probing depth.

^∗^Significant difference between HS and P.

The value of *p* ≤ 0.05 was considered significant.

### 3.2. Analysis of IL‐23 and IL‐17A Expression in GT

GT samples from all study subjects (*n* = 49) were analyzed for IL‐23/IL‐17A molecules and their receptors. However, only representative blots from healthy subjects (*n* = 4) and patients with periodontitis (*n* = 4) are presented for each molecule analyzed.

**Figure 1:**
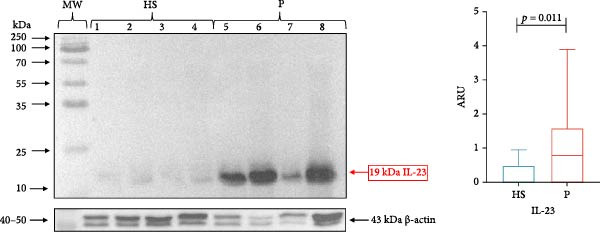
IL‐23 in gingival tissue by western blotting. Representative blot of HS (*n* = 4) and P (*n* = 4) samples. The expression of IL‐23 in gingival tissue was evaluated using the western blotting technique and was expressed as ARU. Representative bands and graphs of IL‐23 (19 kDa) and β‐actin (43 kDa). ARU, area relative units; HS, healthy subjects; MW, molecular weight; P, periodontitis. Medians and the maximum and minimum represent data (Mann–Whitney *U* statistical analysis).

**Figure 2:**
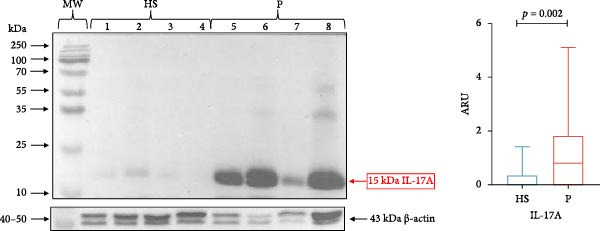
IL‐17A in gingival tissue by western blotting. Representative blot of HS (*n* = 4) and P (*n* = 4) samples. The expression of IL‐17A in gingival tissue was evaluated using the western blotting technique and was expressed as ARU. Representative bands and graphs of IL‐17A (15 kDa) and β‐actin (43 kDa). ARU, area relative units; HS, healthy subjects; MW, molecular weight; P, periodontitis. Medians and the maximum and minimum represent data (Mann–Whitney *U* statistical analysis).

The protein expression data are presented as the median and interquartile range (IQR). IL‐23 (19 kDa) was significantly higher in subjects with periodontitis (median 0.7921, IQR 1.591) compared with healthy subjects (median 0.038, IQR 0.485; *p* = 0.011; Figure 1). Similarly, IL‐17A (15 kDa) expression was significantly higher in periodontitis patients (median 0.789, IQR 1.860) compared with healthy subjects (median 0.00, IQR 0.363; *p* = 0.002; Figure 2).

### 3.3. Analysis of IL‐23R and IL‐17RA Expression in GT

**Figure 3:**
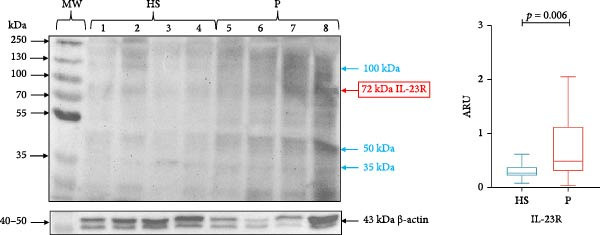
IL‐23R in gingival tissue by western blotting. Representative blot of HS (*n* = 4) and P (*n* = 4) samples. The expression of IL‐23R in gingival tissue was evaluated using the western blotting technique and was expressed as ARU. Representative bands and graphs of IL‐23R (72 kDa) and β‐actin (43kDa). ARU, area relative units; HS, healthy subjects; MW, molecular weight; P, periodontitis. Bands with different molecular weights are indicated in blue. Medians and the maximum and minimum represent data. Mann–Whitney *U* statistical analysis.

**Figure 4:**
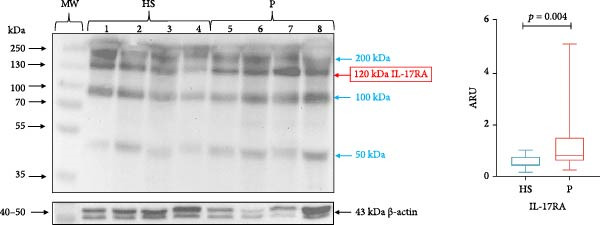
IL‐17RA in gingival tissue by western blotting. Representative blot of HS (*n* = 4) and P(*n* = 4) samples. The expression of IL‐17RA in gingival tissue was evaluated using the western blotting technique and was expressed as ARU. Representative bands and graphs of IL‐17RA (120 kDa) and β‐actin (43 kDa). ARU, area relative units; HS, healthy subjects; MW, molecular weight; P, periodontitis. Bands with different molecular weights are indicated in blue. Medians and the maximum and minimum represent data (Mann–Whitney *U* statistical analysis).

IL‐23R (72 kDa) expression was significantly higher in the periodontitis group (median 0.491, IQR 1.137) compared with healthy subjects (median 0.272, IQR 0.180; *p* = 0.006; Figure 3). Likewise, IL‐17RA (120 kDa) expression was significantly higher in subjects with periodontitis (median 0.804, IQR 0.895) compared with healthy subjects (median 0.479, IQR 0.389; *p* = 0.004; Figure 4).

In addition to detecting the band corresponding to IL‐23R, we observed three other bands of ~35, 50 and 100 kDa. Similarly, when analyzing IL‐17RA, we detected several bands of higher and lower molecular weight than expected (50, 100, and 200 kDa; Figures 3 and 4).

### 3.4. Correlations Between Clinical Parameters of GT and the Expression of IL‐23, IL‐17A, and Their Receptors

**Figure 5:**
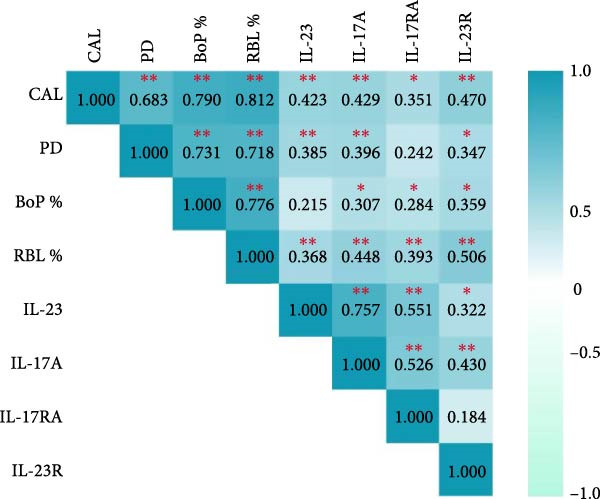
Correlation between IL‐23/IL‐17A axis molecules and IL‐23R and IL‐17RA receptors with clinical parameters in gingival tissue. The Spearman correlation test was used to analyze the values of periodontal clinical parameters and the concentrations of IL‐23, IL‐17A, IL‐23R, and IL‐17RA. BoP%, percentage of bleeding on probing; CAL, clinical attachment loss; IL, interleukin; PD, probing depth; RBL%, radiographic bone loss,  ^∗^ *p* ≤ 0.05,  ^∗∗^ *p* ≤ 0.001.

Spearman correlation coefficients were calculated for the two study groups separately, where it was observed that in the group of healthy subjects there were correlations between: IL‐23R and IL‐17RA [*r* = 0.494 (*p* = 0.012)] and BoP% [*r* = 0.396 (*p* = 0.05)]; as well as between IL‐23 and IL‐17A [*r* = 0.402 (*p* = 0.046)] and PD [*r* = 0.535 (*p* = 0.006)]. Meanwhile, in the group of patients with periodontitis, significant correlations were found between: IL‐23R and IL‐23 [*r* = 0.469 (*p* = 0.021)]; as well as between IL‐17RA and IL‐17A [*r* = 0.713(*p*  < 0.001)] and with IL‐23 [*r* = 0.637 (*p* = 0.001)]. And between IL‐17A and IL‐23 [*r* = 0.871 (*p*  < 0.001)]. However, no significant correlations were found between clinical parameters (data not shown). Finally, correlations between all variables and study subjects were analyzed, and some positive correlations were observed. IL‐23 with CAL, PD, and RBL%; IL‐17A with CAL, PD, BoP%, and RBL%; IL‐23R with CAL, PD, BoP%, and RBL%; and IL‐17RA with CAL, BoP%, and RBL%. In addition, IL‐23 and IL‐17A were positively correlated with each other and with IL‐17RA and IL‐23R (Figure 5).

## 4. Discussion

In the presence of periodontopathogenic bacteria, the host’s immunoinflammatory response is initiated with the activation of various cell lines that release multiple cytokines, including IL‐1β, IL‐6, TNF‐α, and the IL‐23/IL‐17 axis. All these cytokines activate the RANK/RANKL system, which is directly involved in the destruction of tooth support tissues in periodontitis [3, 5, 7, 28, 29].

Regarding the IL‐23/IL‐17 axis, multiple studies have analyzed the levels of this axis in various types of biological samples from periodontitis patients [8–18]. Most of these studies, including our working group, demonstrate an increase in IL‐23, IL‐17A, and IL‐17RA in GT from patients with periodontitis [11, 13–17]. However, we differ in the results obtained for IL‐23R at the protein level [11, 15], compared to those reported by Trumet, who found no significant differences [18], and by Ohyama et al. [16] who found an increase at the gene expression level. In this study, we analyzed the IL‐23/IL‐17A axis and its receptors using the WB technique to elucidate the behavior of IL‐23R, in addition to obtaining more detailed information on the behavior of the molecules that make up this axis.

IL‐23 is a proinflammatory cytokine involved in the maintenance, proliferation, and survival of Th17 cells, which secrete IL‐17 and RANKL [6, 30]. When analyzing IL‐23 expression in GT samples, we found a significant increase in periodontitis patients versus healthy controls; this agrees with the reports by Rodriguez et al. [11] and Ohyama et al. [16], who analyzed this molecule using ELISA, PCR, and immunohistochemistry in various types of biological samples [11, 16]. This increase in IL‐23 could be due to the persistence of *Porphyromonas gingivalis*, which is the predominant bacteria in the advanced stages of periodontitis [31], leading to dysbiosis of periodontal tissues and activation of DCs present in the GT for the constant production and secretion of IL‐23 [4].

IL‐23 binds to its receptor on Th17 cells, releasing RANKL and IL‐17 [30]. IL‐23R is a transmembrane protein expressed by macrophages, DCs, natural killer cells, and Th17 cells [32]. In this study, IL‐23R expression was analyzed, and a significant increase was observed in the periodontitis group compared with the control group. These results agree with the findings reported by Ohyama et al. [16], who analyzed the gene expression of this receptor using PCR. IL‐23 is known to cause IL‐23R overexpression in several cell types [33]; thus, IL‐23 upregulation in periodontitis [11] could cause IL‐23R overexpression in this pathology. We previously reported a decrease in IL‐23R in GT of subjects with periodontitis compared with healthy subjects based on a sandwich ELISA [11, 15]. It is worth mentioning that one of the disadvantages of a sandwich ELISA is that the antigen must show two binding sites, one for the capture antibody and one for the detection antibody [34], so there is a possibility that this technique does not show the actual levels of the target protein (IL‐23R). In contrast, WB relies on binding the antibody to the protein and shows the expected molecular weight and size. These advantages [24] may be why IL‐23R (72 kDa) was increased in GT of patients with periodontitis in this study.

IL‐17A is a proinflammatory cytokine that induces RANKL expression and thus participates in osteoclastogenesis [3, 7]. Several studies have analyzed the role of IL‐17 in various inflammatory diseases, finding elevated levels in patients with rheumatoid arthritis, spondyloarthritis, inflammatory bowel disease, psoriasis, and periodontitis [35, 36]. In the present study, we found a significant increase in IL‐17A expression in periodontitis patients, which agrees with what has been reported by several authors, including our working group, using various techniques [11, 13, 17]. In addition to being synthesized by Th17 cells, neutrophils can also produce IL‐17, the primary cell type in the gingival pocket [37]. Neutrophils participate in inflammation through their extracellular traps (NETs), which induce differentiation of Th17 cells to secrete IL‐17. Thus, the increase in IL‐17 may be due to a positive feedback process whereby neutrophil recruitment and NETosis amplify the Th17 cell response to increase neutrophil recruitment and IL‐17 overexpression in periodontitis [38].

IL‐17RA is a transmembrane protein found in multiple cells and tissues of both the immune and non‐immune systems [39]. We observed a significant increase in IL‐17RA protein expression in the periodontitis group versus the control group. These results coincide with those previously reported by our working group, in which ELISA analyzed this receptor in GT [11, 15]. IL‐17RA is ubiquitously expressed and dynamically modulated in various cells depending on the stimuli they receive [40]. Fibroblasts, the most prevalent cell type connective tissue, are the primary cells that produce IL‐17RA. They are essential for establishing tissue architecture and repairing damaged tissue [41]. Thus, IL‐17RA overexpression in GT of patients with periodontitis might correspond to the receptor expressed on the fibroblast membrane and receptors at the intracellular level [40].

Semi‐quantitatively, using WB, we found overexpression of the IL‐23/IL‐17A axis and its receptors in the GT of patients with periodontitis, which coincides with several authors [11, 13–18], including the expression of IL‐23R, which we had previously found to be decreased by the ELISA method [11, 15]. Another sensitive and specific technique that could be used to quantitatively measure IL‐23/IL‐17 axis molecules in the GT of patients with periodontitis, in addition to clarifying the behavior of IL‐23R is liquid chromatography‐tandem mass spectrometry (LC/MS/MS) [42–44]. However, this technique currently requires a series of steps under optimal conditions to obtain reliable results for the study of cytokines [45–47]. However, although the WB technique used in this study is semi‐quantitative, it provided us with partial data on the expression of the IL‐23/IL‐17A axis.

Regarding Spearman correlation analysis, we observed a significant positive correlation between IL‐23 and IL‐17A with both receptors, which agrees with what has been reported by other authors [8, 48]. This could be because the IL‐23/IL‐17A axis works synergistically to ensure the constant expression of its ILs and receptors. Following this axis, IL‐17A is expressed under the stimulus generated by IL‐23, which is synthesized in the presence of bacteria in the dental plaque [35]. In addition, IL‐23R is stimulated by IL‐23 itself; therefore, when there is an increase in this IL, there is also an increase in the synthesis of its receptor [32], while IL‐17RA is stimulated in the presence of a wide variety of cells and cytokines that participate in inflammatory responses [40].

There were also significant positive correlations between IL‐23, IL‐17A, IL‐23R, and IL‐17RA with the periodontal clinical parameters of CAL and RBL%, which agrees with reports from several authors [13, 14, 49]. These correlations suggest a tight link and clinical importance resulting from higher levels of this axis in periodontal tissue. Thus, the four molecules of the axis may be involved in the progression and severity of periodontitis.

When analyzing the correlations between all variables with the periodontitis group and with the healthy subjects group separately, we found significant positive correlations between cytokines IL‐23 and IL‐17 and their receptors in the periodontitis patient group, suggesting a strong association between molecules in this axis and periodontitis.

Another finding of this study using WB was the detection of bands with unexpected molecular weights for IL‐23R and IL‐17RA. In the case of IL‐23R, three bands (35, 50, and 100 kDa) were detected, different from the canonical form of IL‐23R (72 kDa). In this context, it is known that IL‐23R can occur in isoforms generated by alternative splicing, and 24 isoforms of the IL‐23R subunit have been reported, which can occur as soluble isoforms, premature proteins with short peptides, isoforms without intracellular signaling components, and structurally complete but non‐functional proteins [21, 22, 50]. Another way in which IL‐23R isoforms can be formed is by transmembrane cleavage by a disintegrin and metalloprotease 10 and 17 (ADAM10 and ADAM17) [20], of which ADAM17 has been found to be elevated in patients with periodontitis [51, 52].

Regarding IL‐17RA in this study, we observed three bands of different molecular weight (50, 100, and 200 kDa) than that of the canonical form of IL‐17RA [120–170 kDa). It is known that IL‐17RA isoforms are generated exclusively by alternative splicing [19]. Sohda et al. [19] demonstrated a soluble isoform of IL‐17RA with a molecular weight of 100 kDa.

With the WB technique employed in this study, we could not determine whether these bands detected for IL‐23R and IL‐17RA that differ from the canonical forms correspond to immature forms or isoforms of these proteins. An analysis of N‐glycan removal [53], LC/MS/MS [42, 43], and peptide mapping [54] could help elucidate whether these bands are immature proteins or isoforms of IL‐23R and IL‐17RA in periodontitis.

The study of IL‐23R and IL‐17RA isoforms in periodontitis is important because it has been determined that some IL‐23R gene variants (G149R, R381Q, and V3621) are associated with protection in patients with inflammatory bowel disease, while other variants increase their susceptibility [23]. In this regard, some gene variants of the IL‐23/IL‐17 axis and its receptors have been analyzed in periodontitis, but only associations with IL‐17A and IL‐17F have been found [55]. It should be noted that these studies are at the gene level, but more post‐translational analysis of the IL‐23/IL‐17 axis and its receptors is needed. The identification of isoforms of these proteins could provide a basis for testing them as therapeutic targets or biomarkers in periodontitis, as has been reported in clinical trials for other pathologies (spondylitis, psoriasis, rheumatoid arthritis, and psoriatic arthritis ankylosing) [56]. Importantly, in psoriasis, the use of the monoclonal antibody Brodalumab, which inhibits IL‐17RA and does not allow IL‐17AA, AF, FF, C, and E to bind, has already been approved; this approach blocks the related signaling pathways and functions to improve psoriasis [39, 57]. This finding is relevant because, in the future, some antibodies that block the IL‐23/IL‐17 axis and its receptors [56] could be considered for clinical trials in periodontitis to achieve a beneficial effect in subjects with this disease.

This study has some limitations that must be mentioned. First, because it was a cross‐sectional study, we did not analyze changes in these proteins in the long term to determine whether their production increases or decreases as periodontitis progresses. Therefore, we suggest performing a longitudinal study to follow up and elucidate the behavior of the IL‐23/IL‐17A axis. Second, we did not use a positive control for the expression of IL‐23/IL‐17A axis molecules; so, in the future, we will use a control that strongly expresses the molecules of this axis, such as a cell line or tissue. Third, we did not analyze these molecules using other laboratory techniques to corroborate our results; however, although the WB technique used in this study is semi‐quantitative, it clearly expresses the bands at the corresponding molecular weights of the IL‐23/IL‐17A axis and its receptors (canonical forms) to be semi‐quantified. Finally, WB can indicate different bands corresponding to peptides with different molecular weights, as observed in the case of IL‐23R and IL‐17RA; however, it is impossible to identify the structural nature of these proteins. Therefore, we propose using other techniques to analyze these bands in the future, such as peptide mapping or glycosylation analysis.

## 5. Conclusions

In this study, we were able to quantitatively detect the overexpression of IL‐23, IL‐17A, and their receptors in the GT of patients with periodontitis, which contributes to what has been previously reported by different authors and confirms that the IL‐23/IL‐17A axis is involved in periodontal disease. In the future, this axis could be used as a therapeutic target to inhibit the bone resorption pathway and improve the condition of patients with periodontitis.

## Conflicts of Interest

The authors declare no conflicts of interest.

## Funding

This study was conducted with financial support from the University of Guadalajara and the Mexican Association of Periodontology.

## Data Availability

The datasets used and/or analyzed during the present study are available from the corresponding author upon reasonable request.
